# Supplementary material for: Identification of photoperiod-induced specific miRNAs in the adrenal glands of Sunite sheep (Ovis aries)
Source: Front Vet Sci. 2022 Jul 22;9:888207. doi: 10.3389/fvets.2022.888207 (PMC9354845; doi:10.3389/fvets.2022.888207)
Supplement: Supplementary file 1 [file Data_Sheet_1.ZIP › Supplementary file/miRDeep2 file.zip!/miRDeep2 file/Know_Structure/LP42A3/oar-mir-432.pdf]

miRBase precursor : oar-mir-432
Total read count : 3020
oar-miR-432 read count : 3005
remaining reads : 15

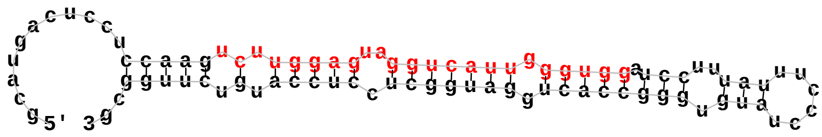

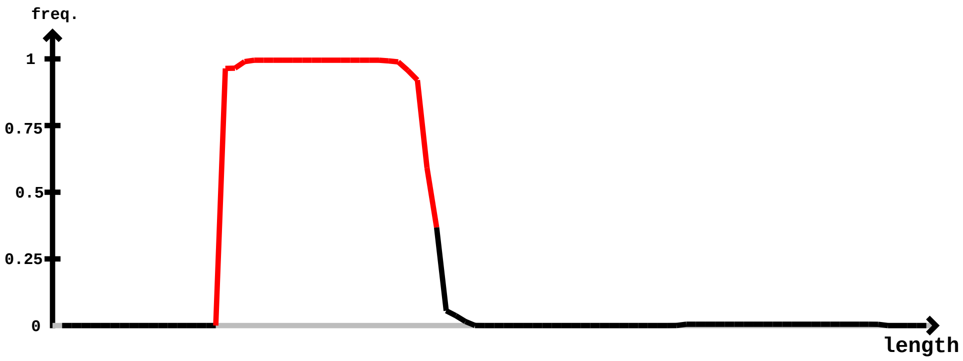


```
                                  oar-miR-432
5'- gcaugacuccuccaagucuuggaguaggucauugggugggauccuuuauuucccuaugugggccacuggauggcuccuccaugucuuggcg   -3'    exp
    ...........(((((.(.(((((..(((((((.(((((.(((..(((......))).)))))))).))))))).))))).).)))))..     reads      mm        sample
    ................ucuuggaguaggucauu.........................................................     8          0         seq
    ................ucuuggaguaggucauug........................................................     11         0         seq
    ................ucuuggaguaggucauugg.......................................................     96         0         seq
    ................ucuuggaguaggucauuggg......................................................     110        0         seq
    ................ucuuggaguaggucauugggu.....................................................     991        0         seq
    ................ucuuggaguaggucauugggug....................................................     675        0         seq
    ................ucuuggaguaggucauugggugg...................................................     942        0         seq
    ................ucuuggaguaggucauugggugga..................................................     50         0         seq
    ................ucuuggaguaggucauuggguggau.................................................     26         0         seq
    ................ucuuggaguaggucauuggguggauc................................................     3          0         seq
    .................cuuggaguaggucauugggu.....................................................     2          0         seq
    .................cuuggaguaggucauugggugg...................................................     1          0         seq
    ..................uuggaguaggucauugggu.....................................................     1          0         seq
    ..................uuggaguaggucauugggugga..................................................     5          0         seq
    ..................uuggaguaggucauuggguggau.................................................     37         0         seq
    ..................uuggaguaggucauuggguggauc................................................     30         0         seq
    ..................uuggaguaggucauuggguggaucc...............................................     1          0         seq
    ...................uggaguaggucauugggugga..................................................     1          0         seq
    ...................uggaguaggucauuggguggau.................................................     3          0         seq
    ...................uggaguaggucauuggguggauc................................................     12         0         seq
    ...............................................................acuggauggcuccuccaugucu.....     1          0         seq
    ................................................................cuggauggcuccuccauguc......     1          0         seq
    ................................................................cuggauggcuccuccaugucu.....     13         0         seq
```
